# Molecular Subtyping and Prognostic Assessment Based on Tumor Mutation Burden in Patients with Lung Adenocarcinomas

**DOI:** 10.3390/ijms20174251

**Published:** 2019-08-30

**Authors:** Changzheng Wang, Han Liang, Cong Lin, Fuqiang Li, Guoyun Xie, Sitan Qiao, Xulian Shi, Jianlian Deng, Xin Zhao, Kui Wu, Xiuqing Zhang

**Affiliations:** 1 School of Future Technology, University of Chinese Academy of Sciences, Beijing 101408, China; 2 BGI-Shenzhen, Shenzhen 518083, China; 3 China National GeneBank, BGI-Shenzhen, Shenzhen 518120, China

**Keywords:** Tumor mutation burden, molecular subtype, lung adenocarcinomas, *RYR2*

## Abstract

The distinct molecular subtypes of lung cancer are defined by monogenic biomarkers, such as *EGFR*, *KRAS*, and *ALK* rearrangement. Tumor mutation burden (TMB) is a potential biomarker for response to immunotherapy, which is one of the measures for genomic instability. The molecular subtyping based on TMB has not been well characterized in lung adenocarcinomas in the Chinese population. Here we performed molecular subtyping based on TMB with the published whole exome sequencing data of 101 lung adenocarcinomas and compared the different features of the classified subtypes, including clinical features, somatic driver genes, and mutational signatures. We found that patients with lower TMB have a longer disease-free survival, and higher TMB is associated with smoking and aging. Analysis of somatic driver genes and mutational signatures demonstrates a significant association between somatic *RYR2* mutations and the subtype with higher TMB. Molecular subtyping based on TMB is a potential prognostic marker for lung adenocarcinoma. Signature 4 and the mutation of *RYR2* are highlighted in the TMB-High group. The mutation of *RYR2* is a significant biomarker associated with high TMB in lung adenocarcinoma.

## 1. Introduction

Lung cancer is the most frequently diagnosed cancer and one of the leading causes of cancerous deaths globally [1]. In the USA in 2019, the estimated new cases of lung cancer were 228,150 and the number of estimated deaths was 142,760 [2]. In China in 2015, the age standardized incidence rate of lung cancer was 733,300, and the estimated mortality was 610,200 [3]. Adenocarcinoma is one of the most common histologic types of lung cancer. Cancer is a complex disease caused by the accumulation of genetic alteration and genome instability [4]. Many endogenous and exogenous factors, such as DNA damage repair inactivation, DNA erroneous replication, microsatellite instability, and carcinogen exposure, can lead to increased somatic mutations. The total number of mutations occurring in a tumor specimen is termed the tumor mutation burden (TMB), which sketches out the status of genomic mutation [5]. Notably, tobacco smoking is the major cause of lung adenocarcinoma, with a high mutation burden [6].

Recent efforts have been made to link genomic mutation profiling to patient characteristics with clinical outcome accelerated precision medicine. There is compelling evidence emerging that TMB is a biomarker of response to immunotherapy, since higher TMB is likely to harbor more neoantigens as targets for activated immune cells. The positive relationship between TMB and response to CTLA-4 and PD-1 inhibition has been shown in melanoma and non-small cell lung cancer [7,8]. The increased frequencies of base pair substitution mutations have been described as one form of genomic instability, which is an evolving hallmark of cancer [9]. TMB, as one of the indications of genomic instability, may also be a biomarker for characterizing patients who could be treated by immunotherapy. The Cancer Genome Atlas (TCGA) project has used whole exome sequencing (WES) to measure TMB across 30 cancer types [10]. However, the study did not comprehensively explore the associations between TMB and lung cancer patient clinical outcomes. Thus, a precise understanding of TMB in lung adenocarcinoma is still lacking.

To this end, we performed a molecular subtyping based on TMB and analyzed the difference between high TMB (TMB-H) and low TMB (TMB-L) from the aspects of clinical features, somatic driver genes, and mutational signatures. Our analysis showed that TMB may be a potential prognostic assessment marker and identified the significant difference between the *RYR2* mutations of TMB-H and TMB-L, which encodes a protein called ryanodine receptor 2 that is responsible for calcium ion transportation within cells. Furthermore, unbiased enrichment analysis also showed that *RYR2* was associated with Signature 4 in TMB-H, which exhibits C > A mutations and is associated with smoking as described in COSMIC (https://cancer.sanger.ac.uk/cosmic/signatures).

## 2. Results

### 2.1. Molecular Subtyping Based on Mutation Burden

**Figure 1:**
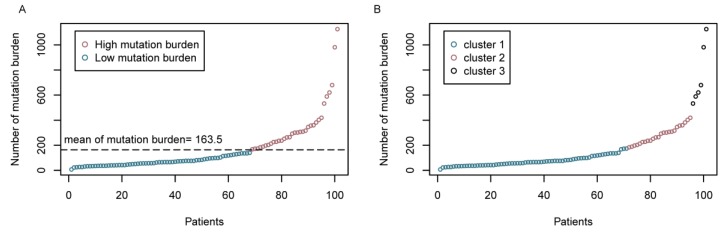
The distribution of TMBs. (**A**) The TMB stratified patients into high mutation burden and low mutation burden types. The threshold is the mean of TMBs of 101 patients, which is equal to 163.5. (**B**) Three clusters divided by optimal univariate k-means clustering and the cluster 1 was strongly similar to the low mutation burden type.

Across the data from LUAD_BGI as described in methods, the values of TMB vary from 7 to 1126, with a mean value of 163.5 (Figure 1A). To determine the critical value of TMB, we took the routine method into account, that population is always divided into groups by mean or median values. For further consideration, we used a dynamic programing optimal univariate k-means clustering method to choose a better grouping threshold between mean and median values. The groups divided by the mean of TMB resemble the cluster result of optimal univariate k-means clustering (Figure 1B). Finally, 33 patients were classified into the TMB-H group, while the other 68 patients were classified into the TMB-L group in accordance with the mean of TMB.

### 2.2. Somatic Driver Genes from Personal Mutation Background

Somatic mutations, including nonsense, missense, splice site, synonymous, frame-shift indel, and nonframe-shift indel, were detected in 33 cases and 68 cases in TMB-H and TMB-L, respectively. To determine the individual mutation profile, we used iCAGES to identify somatic driver genes based on the somatic mutation background and the TMB status of each case as described in the method section. The comprehensive scores of candidate somatic driver genes were evaluated based on their mutational, functional, and drug actionable characteristics.

**Figure 2:**
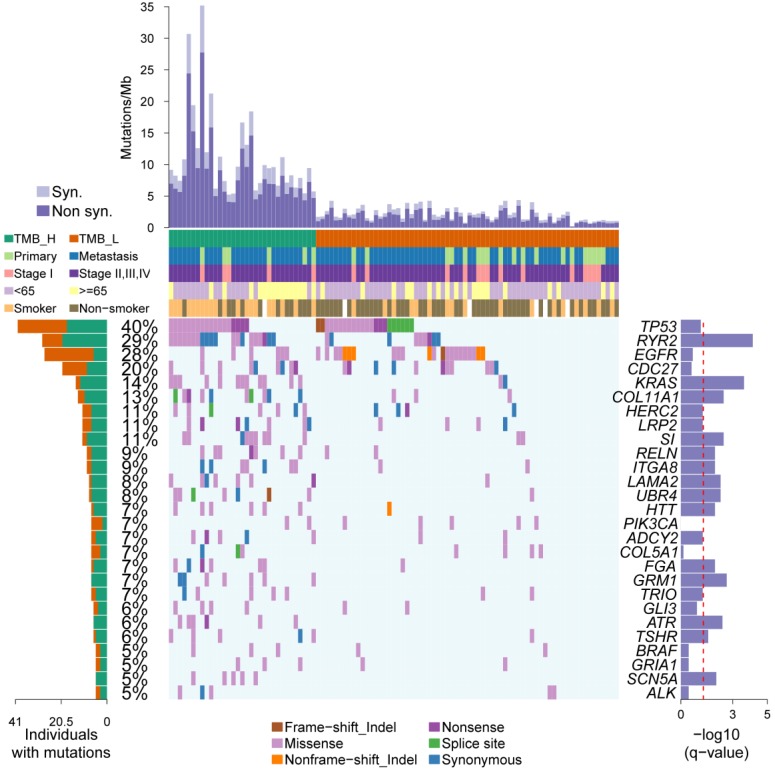
Mutational landscape and the clinical information of 101 patients. The upper side of Figure 2 shows the details of tumor mutation burden (TMB) and the clinical information of each patients. The middle panel of Figure 2 shows the genetic alterations, such as frameshift indel, non-frameshift indel, nonsense mutation, missense mutation, splice site mutation, and synonymous mutation. The left barplot shows the mutational frequency of each gene. The right barplot emphasizes the significant degree of mutation status of each gene, and the *p*-values were adjusted by the Benjamini and Hochberg method (BH). TMB-H, High tumor mutation burden; TMB-L, Low tumor mutation burden.

In the total 101 cases of the LUAD_BGI cohort, 27 somatic driver genes were identified and mutated in at least five cases. In those somatic driver genes, 10 genes, *TP53 (40%), EGFR (28%), CDC27 (20%), KRAS (14%), LAMA2 (8%), PIK3CA (7%), TRIO (7%), ATR (6%), BRAF (5%)*, and *ALK (5%)* were previously reported, whereas the other 17 genes, *RYR2 (29%), COL11A1 (13%), HERC2 (11%), LRP2 (11%), SI (11%), RELN (9%), ITGA8 (9%), UBR4 (8%), HTT (7%), ADCY2 (7%), COL5A1 (7%), FGA (7%), GRM1 (7%), GLI3 (6%), TSHR (6%), GRIA1 (5%)*, and *SCN5A (5%)* were not reported previously (Figure 2). In particular, *TP53, EGFR, RYR2, KRAS*, and *CDC27* were identified as driver genes in more than 10 cases using ICAGEs. Importantly, only *RYR2* was reported for the first time as the mutated cancer driver according to the Cancer Gene Census [11] and IntOGen [12]. 

To determine the driver genes differential between TMB-H and TMB-L, the fisher exact test was performed to figure out the significant driver genes. The mutational status of 14 genes was significantly different between the TMB-H and TMB-L groups and enriched in the TMB-H group. For *EGFR*, *CDC27*, and *PIK3CA* there was only a mutational tendency, indicating a potential feature in the TMB-L group. Among these 14 genes, *RYR2* was the most significant gene with a q value of 6.69 × 10^-5^, indicating the overrepresentation of mutated *RYR2* in the TMB-H group (Figure 2).

**Figure 3:**
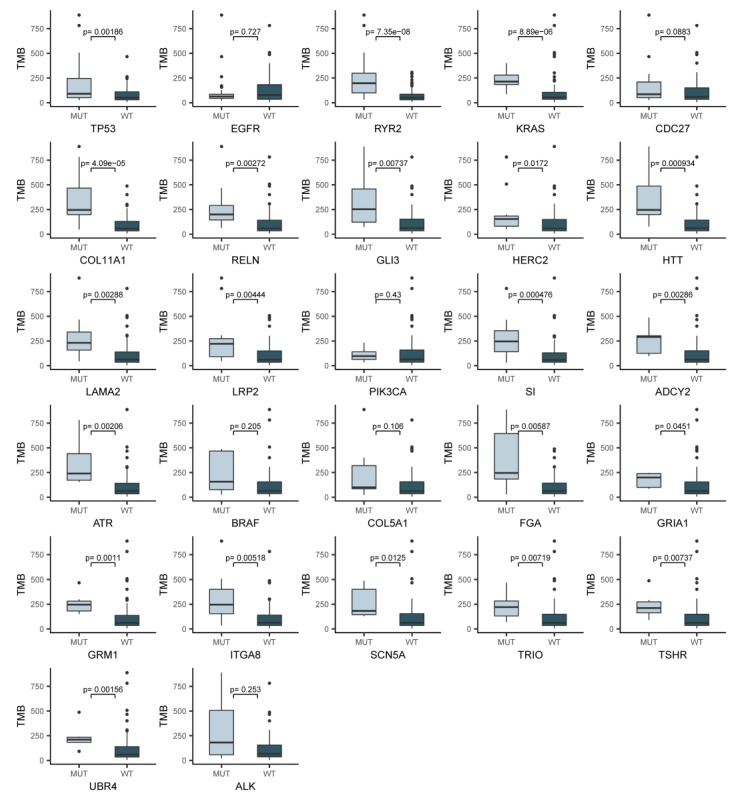
TMB in patients with different mutation status of driver genes. We analyzed TMB status in patients with all 27 different somatic driver genes identified in at least five cases from the total 101 cases of the LUAD_BGI cohort. Notably, TMB is most significantly higher among patients with vs. patients without an alteration in *RYR2*. MUT: mutation; WT: wild type.

For the 27 driver genes, TMB was significantly higher among patients with vs. patients without an alteration in *TP53, RYR2, KRAS, CDC27, COLA11A1, RELN, GLI3, HERC2, HTT, LAMA2, LRP2, SI, ADCY2, ATR, FGA, GRIA1, GRM1, ITGA8, SCN5A, TRIO, TSHR*, and *UBR4*. TMB was most significantly higher among patients with vs. patients without an alteration in *RYR2* (*p*-value = 7.35 × 10^-8^, Figure 3). Patients with the *EGFR* mutation were not associated with high TMB, which was similar to the result that TMB was significantly lower among patients with the *EGFR* mutation [13]. 

### 2.3. An Overview of the Clinical Implications Associated with TMB

**Figure 4:**
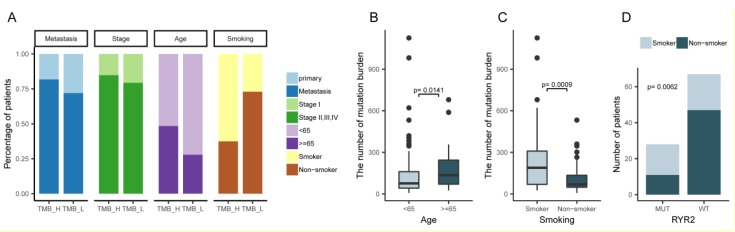
The association between clinical features and TMB. (**A**) The percentage of patients with different clinical features, including age, metastasis status, smoking status, and tumor stage, in TMB-H and TMB-L groups. (**B**) The association between TMB and age. (**C**) The association between TMB and smoking history. (**D**) The association between *RYR2* mutational status and smoking history.

**Figure 5:**
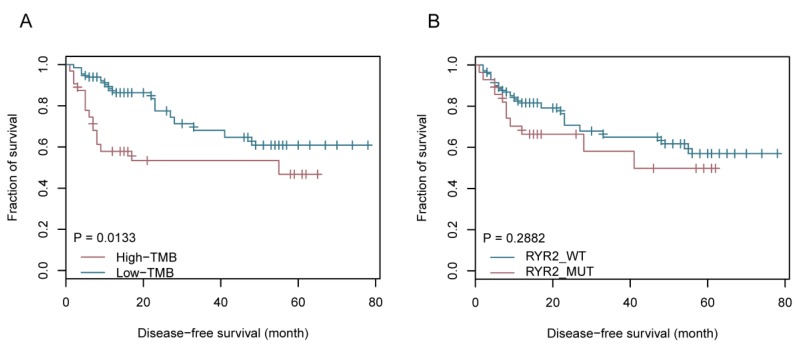
Prognostic significance of molecular subtyping based on TMB and *RYR2* mutational status. (**A**) Patients with low TMB have a longer disease-free survival. (**B**) Patients without *RYR2* mutation have a tendency to show a longer disease-free survival.

Next, we integrated clinical features of the 101 cases into the aforementioned molecular subtypes to explore whether the clinical features were associated with the two TMB groups. Among the clinical factors, such as age, metastasis status, smoking history, and tumor stage, only smoking history and age were associated with TMB levels. There were significantly more patients over the age of 65, or with smoking history, in the TMB-H group with a *p*-value of 0.0006 and 0.042, respectively (Figure 4A). Higher percentages of patients who were under worse status, such as late tumor stage and tumor metastasis condition, were also observed in the TMB-H group, indicating that these clinical factors may be associated with higher TMB levels to some degree (Figure 4A). Further analysis showed a disparity in the number of TMB between the age >=65 and age <65 groups, and between the smoker and non-smoker groups, demonstrating that the accumulation of TMB in the age >=65 and the smoker group is much higher than the age <65 group (*p*-value = 0.0141) and the non-smoker group (*p*-value = 0.0009) (Figure 4B,C). Fisher exact tests showed a positive association between the *RYR2* mutation and smoking history (*p*-value = 0.0062, Figure 4D). Further, Kaplan–Meier survival analysis showed a significantly longer disease-free survival (DFS) in patients with low TMB levels after surgery (*p*-value = 0.0133, Figure 5A); even though the *p*-value is not significant in the survival analysis of *RYR2* mutational status, patients without the *RYR2* mutation showed the tendency of longer DFS (Figure 5B). This result suggests that TMB is potentially associated with favorable DFS in lung adenocarcinoma.

### 2.4. Mutation Signature Analysis of Lung Adenocarcinoma in TMB Subtypes

To understand the mutation accumulation processes during lung adenocarcinoma, we performed mutational signature analysis on these 101 cases. We applied a Bayesian NMF algorithm to mutation counts, stratified by 96 trinucleotide mutational contexts to infer (i) the number of operating mutational processes, (ii) their signatures (96 normalized weights per process), and (iii) the pattern of each signature in lung adenocarcinoma (the estimated number of mutations associated with each signature) [14].

**Figure 6:**
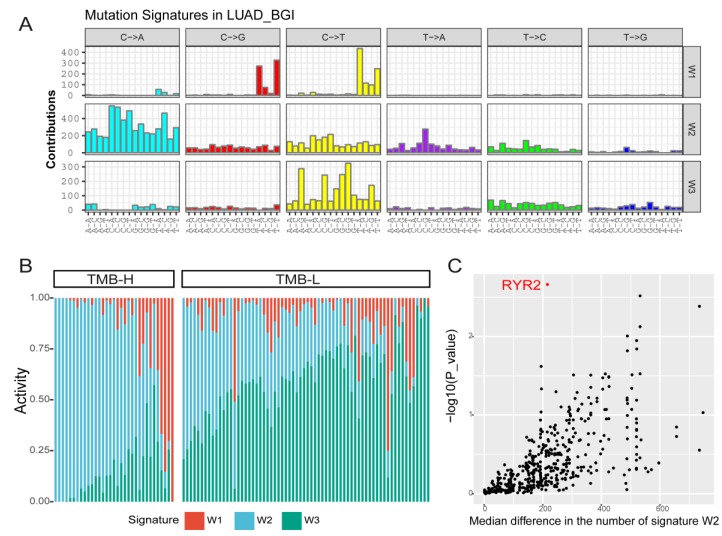
Mutational signature analysis of 101 patients. (**A**) A Bayesian NMF algorithm was applied to identify signatures from the matrix of mutation counts according to 96 types of trinucleotide motifs. Three mutational signatures are identified. (**B**) The distribution of three mutational signatures in the TMB-H and TMB-L groups. Signature W2 is predominant in the TMB-H group. (**C**) Mutation enrichment analysis identifies the association between *RYR2* mutations and pattern of Signature W2. *RYR2* was the top significant gene.

Our analysis identified three mutational signatures in the LUAD-BGI cohort, and we compared them with those applied by the Sanger Institute, which are described in the COSMIC database (http://cancer.sanger.ac.uk). Signature W1, characterized by C > G transversions and C > T transitions at TpCp[A/C/G/T] motifs, corresponds to COSMIC Signature 2 (cosine similarity of 0.85), which is also attributed to the activity of the AID/APOBEC family of cytidine deaminases (Figure 6A). Signature W2, characterized by C > A transversions at a broad spectrum of bases context, closely resembles COSMIC Signature 4 (cosine similarity of 0.96). COSMIC Signature 4 is associated with smoking, and its profile is similar to the mutational pattern observed in experimental systems exposed to tobacco carcinogens (e.g., benzo[a]pyrene). Signature W3, characterized by C > T transitions at [A/C/G]pCpG motifs, corresponds to COSMIC Signature 6 (cosine similarity of 0.91) that is associated with defective DNA mismatch repair.

The TMB-H and TMB-L groups were clearly distinguished by mutation signature spectrums, especially the pattern of Signature 4 (Figure 6B, Wilcoxon rank-sum test, *p*-value = 9.984 × 10^-9^), suggesting an underlying influence of mutation signature on molecular subtyping.

We next performed signature enrichment analysis to further characterize mutated genes associated with Signature 4. We compared the pattern of Signature 4 in tumors that harbored a nonsynonymous mutation in the gene and tumors that did not cross the LUAD_BGI cohort. To exclude the noise resulting from TMB, we assessed the significance level using a permutation-based method that controls the TMB in each sample [14]. We found that *RYR2* was the most significant gene associated with Signature 4 (Figure 6C).

### 2.5. Profile of Gene Expression Level in TMB Subtypes

**Figure 7:**
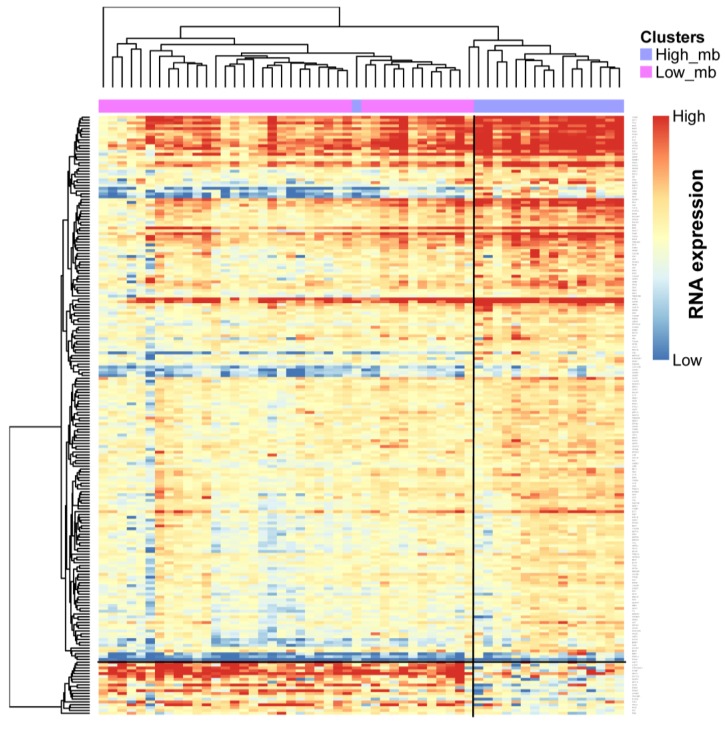
Unsupervised hierarchical clustering of 56 patients identifies two mRNA clusters/groups. The TMB feature is indicated by the annotation bars above the heatmap.

Unsupervised hierarchical clustering analysis was performed, based on 212 differential genes in 56 samples, 19 of which were from TMB-H and 37 samples from TMB-L. We identified two mRNA groups with distinct TMB characteristics, which corresponded to TMB subtypes, with only one sample of TMB-H clustered to TMB-L (Figure 7). We also found a gene cluster with 19 genes (*FMN1, FHIT, FDXR, HHLA2, F2RL1, PLXNA2, TNKS1BP1, CACNB1, SPINK5, BTBD9, CRYM, MPV17L, SH3RF2, SULT1C2, ABCC3, FCGBP, ST6GALNAC1, CLDN1, GDF15*) that were down-regulated in TMB-H. Using gene set enrichment analysis, we identified that these genes were significantly enriched (FDR < 0.05), with a gene set that was down-regulated in epidermis after Ultraviolet B irradiation associated with mutations accumulation [15].

## 3. Discussion

We constructed a molecular subtype model based on the TMB status and showed that the patients from the LUAD_BGI cohort can be further stratified into TMB-H and TMB-L subtypes by the mean of population TMBs. This observation was similar to the result from the analysis using the dynamic programing optimal univariate k-means clustering method. Patients in the subset of TMB-L had a better prognosis, as evidenced by a longer DFS, compared with patients in the TMB-H subset. TMB as a genomic marker of prognosis and a predictor of treatment response has been reported in ovarian cancer [16]. In this study, for the first time, we demonstrated that lower TMB predicts more favorable DFS in patients with lung adenocarcinoma in the Chinese population. The molecular subsets of TMB-L and TMB-H could assist the assessment of prognosis for further clinical research of lung cancer. Exploring the clinical features associated with TMB is also essential to understand and characterize the implications of the molecular subtypes based on TMB. Higher TMB was significantly associated with the group of patients older than 65, or with smoking history, which were the risk factors of carcinogenesis in a common sense [17]. The TMB-H group consisted of patients associated with cancer risk factors, such as smoking and aging, and are in accord with the result of unfavorable prognosis. Non-smokers had a significantly better prognosis compared to current smokers, and older age was an independent predictor of early mortality [18]. A systematic review with meta-analysis also provided the evidence that smoking cessation after diagnosis of early stage lung cancer improves prognostic outcomes [19]. There were mutually verifying relationships between the results of survival analysis and clinical features association analysis [20,21]. Compared with other genome-based molecular subtyping studies [22,23,24], this finding highlighted the importance of molecular subtyping based on TMB in clinical utility.

However, recent research has demonstrated that high TMB was associated with a better prognosis in patients with resected non-small-cell lung cancer (NSCLC), while lung cancer-specific survival with adjuvant chemotherapy was more significant in patients with low TMB [25]. We speculate that one of the possible explanations could be the different regions of genes found while calculating the TMB result in the different associations between TMB and prognosis, and, in our study, TMB was calculated by nonsynonymous mutations in whole exon regions, not targeted panels. These observations and underlying mechanisms between TMB and prognosis should be confirmed by further studies.

In addition to deciphering the association of TMB with clinical features, we also analyzed mutational signatures in the TMB subtypes, and we found that the Signature 4 pattern was increased in the TMB-H group by using signature enrichment analysis as shown in Figure 6. Noteworthy, the Signature 4 pattern is found to be associated with smoking only in the cancer types in which tobacco smoking increases risk and mainly in those derived from epithelia directly exposed to tobacco smoke [26]. Signatures from polycyclic aromatic hydrocarbon, which is the mutagenic component of tobacco smoke, show greatest similarity to Signature 4 [27]. Patients with mutational landscapes dominated by C > A transversions, such as Signature 4, were likely to benefit from immune checkpoint blockade, and this smoking signature was predictive of immune checkpoint blockade response, which has a positive relationship with TMB-H [8,28]. The inter relations of TMB with smoking demonstrated that the TMB triggered by tobacco exposure might be detected as the profile of Signature 4 in TMB-H.

Additionally, we identified an association between Signature 4 and somatic mutations in *RYR2*, which was also mutated significantly in TMB-H, providing important insights for molecular subtyping based on TMB. *RYR2*, ryanodine receptor 2, encodes a ryanodine receptor found in cardiac muscle sarcoplasmic reticulum and induces the release of calcium from the sarcoplasmic reticulum into the cytosol [29]. We found a high proportion of synonymous mutations in *RYR2* (38%, 11/29) and an insignificant tendency for longer DFS in patients without *RYR2* mutations. Considering the association between *RYR2* mutational status and smoking history, mutations in *RYR2* might be accumulated during the history of tobacco exposure and play roles as passenger mutations to impact the process of lung adenocarcinoma. Passenger mutations that fall within coding regions or functional elements could cause potential deleterious effects on cancer development [30]. Nicotine, the major component of tobacco smoke, upregulates *RYR2* via enhanced network Ca^2+^ activity [31]. The association between the *RYR2* mutation and smoking might provide a plausible explanation for the association between the *RYR2* mutation and the Signature 4 pattern. Mutations in *RYR2* are frequently reported in ventricular tachycardia [32,33] and arrhythmogenic right ventricular dysplasia type 2 [34]. Although *RYR2* is known to be associated with heart disease, *RYR2* as one of the components of a calcium channel that also influences calcium signaling in airway smooth muscle cells [35,36,37]. Recent research demonstrated the association between *RYR2* and asthma by genome-wide analysis [38]. Also, cigarette smoke exposure causes down-regulation of ryanodine receptors in mice, and airway smooth muscle and small airway contraction, which is a major site of airflow limitation in chronic obstructive pulmonary disease (COPD) [39]. Therefore, mutations in *RYR2* may result in the alteration of airway smooth muscle and asthma, which is a possible risk factor for lung cancer [40,41]. This is a possible underlying mechanism by which the association between *RYR2* and lung cancer could arise.

Together, we performed a comprehensive analysis on TMB in lung adenocarcinoma, and our results suggests that molecular subtyping based on TMB could be considered as a prognostic marker. Also, Signature 4 and the significance of mutation in *RYR2* are highlighted in the TMB-H group. Further studies are needed to characterize the mechanism underlying mutation of *RYR2* in lung cancer and to explore potential relationships between *RYR2* and high TMB.

## 4. Materials and Methods

### 4.1. Lung Adenocarcinoma Genome Data

All the somatic mutations data, transcriptome sequencing data, and clinical information were downloaded and collected from previously published Chinese lung adenocarcinoma projects [42]. The clinical information was provided as Supplementary Data. The data reported in this study are also available in the CNGB Nucleotide Sequence Archive (CNSA: https://db.cngb.org/cnsa; accession number CNP0000249). This project hereinafter will be referred to as LUAD_BGI. The primary tumor specimens were obtained from patients with lung adenocarcinoma who underwent surgical resection. None of the patients were subjected to chemotherapy or radiotherapy before surgery.

### 4.2. Mutation Burden Cluster

Tumor mutational burden (TMB) is defined by the number of somatic mutations per genome area for target sequencing [43]; for WES data, TMB is defined by the number of somatic mutations [44]. Considering the instability of outliers in the LUAD_BGI somatic mutation data, we used dynamic programming to cluster univariant data given by the mutation burden of all the samples into optimal groups. This algorithm guarantees the optimality of clustering based on the minimum within-cluster sums of squares to give the optimal number of clusters k. To test the cluster number k, a range from 1 to 9 is provided for k, and the optimal number of clusters was determined by Bayesian information criterion. The R package, Ckmeans.1d.dp [45], was performed for selecting the optimal number of clusters k.

### 4.3. Identification of Somatic Driver Genes

We predicted somatic driver genes using iCAGES [46], an efficient tool to search cancer driver genes, based on the somatic mutation data of each case. Three layers of analysis steps were executed with the iCAGES tool. In the first layer, a support vector machine (SVM) was trained on somatic single nucleotide variants from COSMIC and Uniport databases to calculate the SVM score and evaluate the driver mutation potential of each mutation. The second layer weighed each mutation through integrating candidate mutation from the first layer with prior biological knowledge on genetic-phenotypic association information. The third layer prioritized for candidate driver mutation with corresponding drug activity scores from the PubChem database. For the identified somatic driver genes, they were selected from the iCAGES candidate gene list if the gene was regarded as a driver gene in at least five cases.

### 4.4. Mutation Signature Analysis

The mutation signature analysis is a procedure for deconvoluting cancer somatic mutation counts, stratified by mutation contexts or biologically meaningful subgroups, into a set of characteristic signatures and inferring the pattern of each of the discovered signatures across samples. All single nucleotide variants (SNVs) were classified into 96 possible mutation categories based on the six base substitutions (C > A, C > G, C > T, T > A, T > C and T > G) according to complementary base-pairing and 16 possible combinations of neighboring bases within the trinucleotide sequence context. We used Signature Analyzer, which uses a Bayesian variant of NMF and was recently applied to several cancer genome projects [14,47]. To compare the values of the signature pattern from the TBM-L and TMB-H groups, the Wilcoxon rank-sum test was used to identify mutation signature difference.

### 4.5. Permutation Test of Signature Enrichment Analysis

The correlation between the pattern of signatures and the overall TMB could confuse the relationship of genes and the associated signatures. A traditional statistical test, which compares the pattern of signatures among samples where the gene is wild type versus mutant for searching discrepant genes, overestimates the significance of *p* values related to TMB. The samples with a higher TMB prefer to generate more mutations, which confound the statistical test power.

We controlled both the gene-specific and sample-specific mutation counts during the random permutation process to generate a gene x sample binary mutation matrix, following the ‘Curveball algorithm’ described by Strona et al. [48]. We used the one-tailed Wilcoxon rank-sum test to compare the signature pattern of wild and mutant samples of a given gene. The statistic T_observed_ was a Wilcoxon statistic of actually observed data; the statistic T^r^_random_ was a Wilcoxon statistic of every permuted binary mutation matrix, where r = 1, 2, …, 200,000, the total number of permutation times. The finial *p* value of a given gene was the fraction of T^r^_random_ more extreme than T_observed_. This permutation test was followed by the description from Kim et al. [14].

### 4.6. Gene Expression Analysis

The gene expression level was calculated by the RPKM method [49] from 56 lung adenocarcinomas in LUAD_BGI. Differential gene expression was determined by edgeR, a Bioconductor package [50], between high TMB and low TMB groups.

## Supplementary Materials

The following are available online at https://www.mdpi.com/1422-0067/20/17/4251/s1.

## Abbreviations

TMB	Tumor mutation burden
TMB-H	High tumor mutation burden
TMB-L	Low tumor mutation burden
